# Optimized PD-L1 scoring of gastric cancer

**DOI:** 10.1007/s10120-021-01195-4

**Published:** 2021-05-05

**Authors:** Birgid Schoemig-Markiefka, Jana Eschbach, Andreas H. Scheel, Aylin Pamuk, Josef Rueschoff, Thomas Zander, Reinhard Buettner, Wolfgang Schroeder, Christiane J. Bruns, Heike Loeser, Hakan Alakus, Alexander Quaas

**Affiliations:** 1 Department of General, Visceral, Cancer and Transplantation Surgery, University Hospital Cologne, Cologne, Germany; 2 Institute of Pathology, University Hospital Cologne, Kerpener Str. 62, 50937 Cologne, Germany; 3 Department i of Internal Medicine, Center for Integrated Oncology Aachen Bonn Cologne Duesseldorf, Gastrointestinal Cancer Group Cologne GCGC, University of Cologne, Cologne, Germany; 4 Institute of Pathology Nordhessen, Kassel, Germany; 5 Targos Molecular Pathology GmbH, Kassel, Germany

**Keywords:** PD-L1, Biomarker, Checkpoint inhibition, Gastric cancer, Endoscopically obtained, Biopsies

## Abstract

**Background:**

PD-1/PD-L1-Immunotherapy has been approved for gastric carcinoma. PD-L1 assessment by immunohistochemistry is the principle biomarker. Are biopsies able to map the actual PD-L1 status of the entire tumor?

**Methods:**

Whole tumor slides of 56 gastric carcinoma were analyzed to determine the distribution of PD-L1 positive cells in the entire tumor areas. Tissue micro arrays with four cores of the tumor surface, which represents the endoscopically accessible biopsy zone, were built from the same tumors. The PD-L1 CPS value was determined separately for each core. Preoperative diagnostic biopsies were available for 22 of the tumors. PD-L1 prevalence, sensitivity and specificity were analyzed using the whole tumor slides as reference scores. Molecular subtyping was performed and related to the PD-L1 status.

**Results:**

27.3% of cases were PD-L1 negative (CPS < 1), 43.6% showed low PD-L1 expression (CPS ≥ 1 to < 5), 12.7% moderate (CPS ≥ 5 to < 10) and 16.4% strong expression (CPS ≥ 10).

The biopsies showed best test characteristics if four surface biopsies were analyzed combined, i.e., the CPS was calculated across all four biopsies. The prevalence showed a distribution similar to the resection specimens, sensitivity was 0.73 and specificity 1.0. Using fewer surface biopsies decreased sensitivity and specificity and caused false-negative classifications. Compared to the TMAs, the preoperative biopsies showed reduced sensitivity (0.412).

**Conclusions:**

This is the first comprehensive study to optimize PD-L1 assessment in gastric cancer using endoscopically available tissue. The obtained PD-L1 prevalence is consistent with data of current clinical studies. Calculation of the test characteristics shows that surface biopsies can be indicative of the true PD-L1 status based on the resection specimen. However, an adequate number of biopsies is required. In this study, *n* = 4 biopsies yielded best results.

**Supplementary Information:**

The online version contains supplementary material available at 10.1007/s10120-021-01195-4.

## Introduction

Immune checkpoint inhibition plays a decisive role in modern oncology. The PD-1 (programmed cell death 1) receptor and its ligand PD-L1 are physiologically involved in immunomodulation [1]. Many malignant tumors show aberrant PD-L1 expression on the carcinoma cells and/or tumor-associated immune cells [2]. PD-L1 overexpression is associated with interferon gama signaling in the stroma [3], WNT/β-catenin and PIK3CA/PTEN signaling [4], Epstein-Barr Virus infection [5] and microsatellite instability (MSI) [2]. Clinical trials on PD-1 inhibition in gastric cancer (GC) were successful (CheckMate 649; Keynote-61) and anti-PD-1 antibodies have been approved for gastric adenocarcinoma [6] and esophageal squamous cell carcinoma. [7], 8] PD-L1 immunohistochemistry (IHC) is currently the principle biomarker for immunotherapy and is predictive for both anti-PD-1 and anti-PD-L1 treatments. More biomarkers such as tumor mutational burden (TMB) and RNA-based expression analysis are investigated but not yet clinically employed [2, 4].

In GC, the EBV-positive and the MSI molecular subtypes are characterized by strong overexpression of PD-L1 in tumor, stromal and immune cells [9, 10]. Tumors with overexpression of PD-L1 were associated with a better prognosis analyzed in a group of Western patients while former studies with Asian patients showed a worse prognostic effect. The prognostic difference has been related to different genetic signatures [11].

A growing number of studies have been conducted to investigate immunotherapy-options for gastric cancer patients. Some studies tested single agent regimes, some ongoing trials evaluate combination approaches with chemotherapy and/or molecular targeted agents in different disease settings. Most studies test PD-L1 IHC as predictive biomarker, e.g., [12–14]. The most common type of interpretation is the IHC combined positive score (CPS) [15] which evaluates PD-L1 expression on carcinoma cells and tumor-associated immune cells. Different cut-off values are investigated (CPS 1, 5, 10). Generally speaking, a higher cut-off reduces the number of positive cases but increases clinical benefit.

GC is usually diagnosed by small endoscopic biopsies. IHC for Her2 and PD-L1 is often performed on the biopsy specimens, in particular in the neoadjuvant setting. PD-L1 expression shows spatial heterogeneity in most GC cases, yet little is known about the validity of PD-L1 scoring on GC biopsies. The aim of this study was to optimize PD-L1 assessment in GC. Four superficial and four deep biopsies were compared to matched resection specimens. Estimated prevalence, sensitivity and specificity were calculated for different conditions and an optimal procedure was determined.

## Methods

### Patients’ samples

Formalin-fixed, paraffin embedded (FFPE) tissue from *n* = 56 patients with gastric adenocarcinoma was analyzed including 56 resection specimens and 22 preoperative biopsies. Biomaterials and clinical data were used in agreement with the guidelines of the local ethics committee. The usage of the FFPE materials was consented. Patients were treated with primary surgical resection between 2016 and 2018 at the Department of General, Visceral and Cancer Surgery, University of Cologne, Germany.

### Tissue microarray construction

**Fig. 1:**
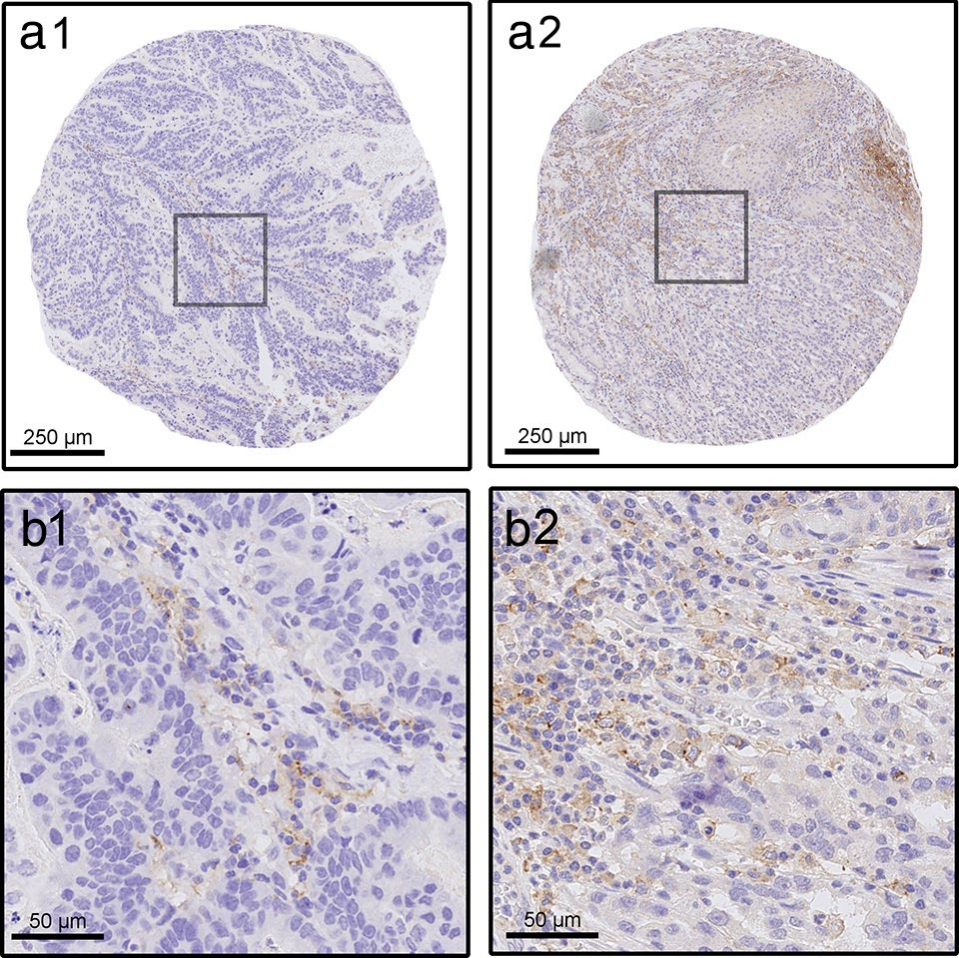
PD-L1 immunohistochemistry. Examples of two tissue-microarray cores with PD-L1 immunohistochemistry (IHC) showing focal expression in tumor-associated immune cells (**a**) and widespread expression (**b**). Overview of the cores (1), magnified detail (2)

Tissue microarrays (TMAs) of the *n* = 56 gastric adenocarcinoma were constructed as previously described [16, 17]. Two intratumoral regions per case were identified on H&E stained slides. Each region was samples by four TMA cores with a diameter of 1.2 mm and an area of 1.13 mm^2^. (Supplementary Information (SI Fig. 1). The surface region represents the superficial, endoscopically accessible biopsy zone. The deep region represents the invasive front of the carcinoma, which is endoscopically unreachable. The regions were selected to contain viable tumor-cell formations and their adjacent stroma. Areas with alterations that are known to infere with IHC were excluded, i.e., necrotic areas, fibrinous exsudate, detritus and areas with artificial fragmentation. TMA cores containing tonsil tissue were included as internal control and for spatial orientation. Example photomicrographs show representative TMA cores (Fig. 1) and one TMA slide (SI Fig. 2).

### Determination of molecular tumor subtypes according to TCGA

Molecular subtyping was performed according to the current WHO recommendations of 2019. The EBV subtype was determined using the specific RNA in-situ test “EBER” (PB0589, Leica, Germany). The MSI subtype was determined by IHC for mismatch repair deficiency (MMR) as recommended (mouse monoclonal primary antibodies: MLH1 clone M1, MSH2 clone G219-1129, PMS2 clone A16-4; rabbit monoclonal antibody: MSH6, clone SP93. All clones by Roche Diagnostics, Switzerland). IHC results indicative of MMR deficiency (d-MMR) were confirmed by PCR using the Bethesda panel. All d-MMR tumors were highly microsatellite-instable (MSI-H).

The GS and CIN subtype, on the other hand, are less clearly definable. In the following, we refer to the GS subtype as “diffuse/CDH-type” if these tumors show a poorly cohesive and/or signet-ring cell growth pattern (= diffuse type according to Lauren). The IHC loss of E-cadherin (mouse monoclonal antibody, clone NCH-38, Dako/Agilent, USA) with a concomitant diffuse growth pattern and the absence of MSI or EBV were further arguments for the assignment to this subtype. The CIN subtype corresponded to all tumors that could not be assigned to the defined other subgroups (EBV, MSI, “diffuse/CDH-type”). These tumors typically showed the following characteristics: intestinal (glandular) morphology, TP53 alteration as determined by immunohistochemistry (mouse monoclonal antibody, clone DO-7, Dako/Agilent, USA) and more frequent Her2 alteration as determined by immunohistochemistry (rabbit monoclonal antibody, clone 4B5, Roche, Switzerland) and fluorescence in-situ hybridization for Her2/neu (Zytolight SPEC ERBB2/CEN 17 Dual Probe Kit; Zytomed Systems GmbH, Germany). In the following, we therefore refer to this subtype as the “intestinal type”.

### PD-L1 immunohistochemistry and scoring

PD-L1 immunohistochemistry was performed by a laboratory developed test (LDT) using primary antibody clone E1L3N (Cell Signaling Technology, Danvers, USA) at 1:400 dilution on the Leica Bond Max staining platform (Leica Biotechnologies, Wetzlar, Germany). Antigen-retrieval was achieved by heat-induced antigen-retrieval with citrate buffer. Detection was done by the Bond Polymer Refine system (Leica Biotechnologies). The PD-L1 LDT was calibrated to match the staining patterns of the Agilent/Dako 22C3 pharmDx assay and validated by external quality assessment (QuIP GmbH, Berlin, Germany). Tonsil tissue was used as on-slide control on all PD-L1 IHC stainings. All samples in this study (resection specimens, diagnostic biopsies and TMAs) were stained using the same IHC protocol.

Scoring was done according to the Agilent/Dako 22C3 pharmDx assay for gastric cancer guidelines (Agilent Technologies, Santa Clara, USA) and the CPS was quantified as described by the manufacturer. Samples were independently scored by four board-certified and PD-L1 IHC trained pathologists and a consensus-score was calculated for each sample. Interobserver concordance was quantified by Cohen’s weighted kappa coefficient (SI Table 1). Pairwise comparisons yielded kappa values of 0.42–0.74 for the biopsies ('substantial' according to the interpretation by Landis and Koch) [18] and 0.3–0.67 for the resection specimens (‘moderate’). The consensus scores showed kappa values of 0.54–0.84 for the biopsies and 0.56–0.74 for the resection specimens (‘substantial’).

### Statistical analyses

Data analyses were performed using Microsoft Excel version 2016 and ‘R’ statistical programming language version 3.6.2. ‘R’ package ‘psy’ was used for interobserver concordance analyses.

PD-L1 IHC scores of the resection specimens were considered as representative of the true PD-L1 status of respective tumors. The scores of the resection specimens were used as reference values in subsequent calculations of specificity and sensitivity.

The four superficial and the four deep tissue samples were integrated by calculating the average CPS value using the arithmetic mean. Integration using the maximum CPS value was tested, i.e., the highest CPS value of any of the four samples was used to classify the respective case. For comparisons of PD-L1 expression in the samples and resection specimens, the CPS was categorized into a four-step score (0–3) based on clinical relevant thresholds: 0 (CPS < 1), 1 (CPS 1–4), 2 (CPS 5–9), 3 (CPS ≥ 10). In the analyses of one, two or three biopsies per case, all possible permutations were incorporated.

## Results

### Tumor- und patient characteristics

The PD-L1 status was determined by immunohistochemistry using the combined-positive-score (CPS). First, whole tumor slides in *n* = 56 patients with gastric adenocarcinoma were tested. In *n* = 22 patients, preoperative diagnostic biopsies were available and also tested. The patients were treated with primary surgery without neoadjuvant chemotherapy. Second, corresponding TMAs of the same cases were tested that mimic tumor biopsies in size and location (Fig. 1, SI Figs. 2, 3) 58.9% of the patients were men and 91.1% were over 50 years old at the time of surgery. The majority of tumors were located proximally or in the gastric corpus (71.4%). The molecular tumor subtypes according to TCGA were dominated by the “intestinal type” (80.4%), followed by “diffuse/CDH-type” (10.7%), microsatellite instable (MSI, 5.4%) and Epstein-Barr virus-associated subtype (EBV, 3.6%) (Table 1).

**Table 1:** Patients’ characteristics

		*n*	%
Sex	Male	33	58.9
	Female	23	41.1
Age	< = 50	5	8.9
	> 50	51	91.1
Localisation	Proximal	24	42.9
	Corpus	18	32.1
	Distal	12	21.4
	Stump	2	3.6
pT	pT1	14	25.9
	pT2	4	7.4
	pT3	21	38.9
	pT4	15	27.8
	Missing	2	
pN	pN0	20	36.4
	pN1	6	10.9
	pN2	12	21.8
	pN3	17	30.9
	Missing	1	
UICC	UICC 1	15	27.3
	UICC 2	10	18.2
	UICC 3	24	43.6
	UICC 4	6	10.9
	Missing		
TCGA	CIN	45	80.4
	GS	6	10.7
	MSI	3	5.4
	EBV	2	3.6

### Whole tumor areas

**Fig. 2:**
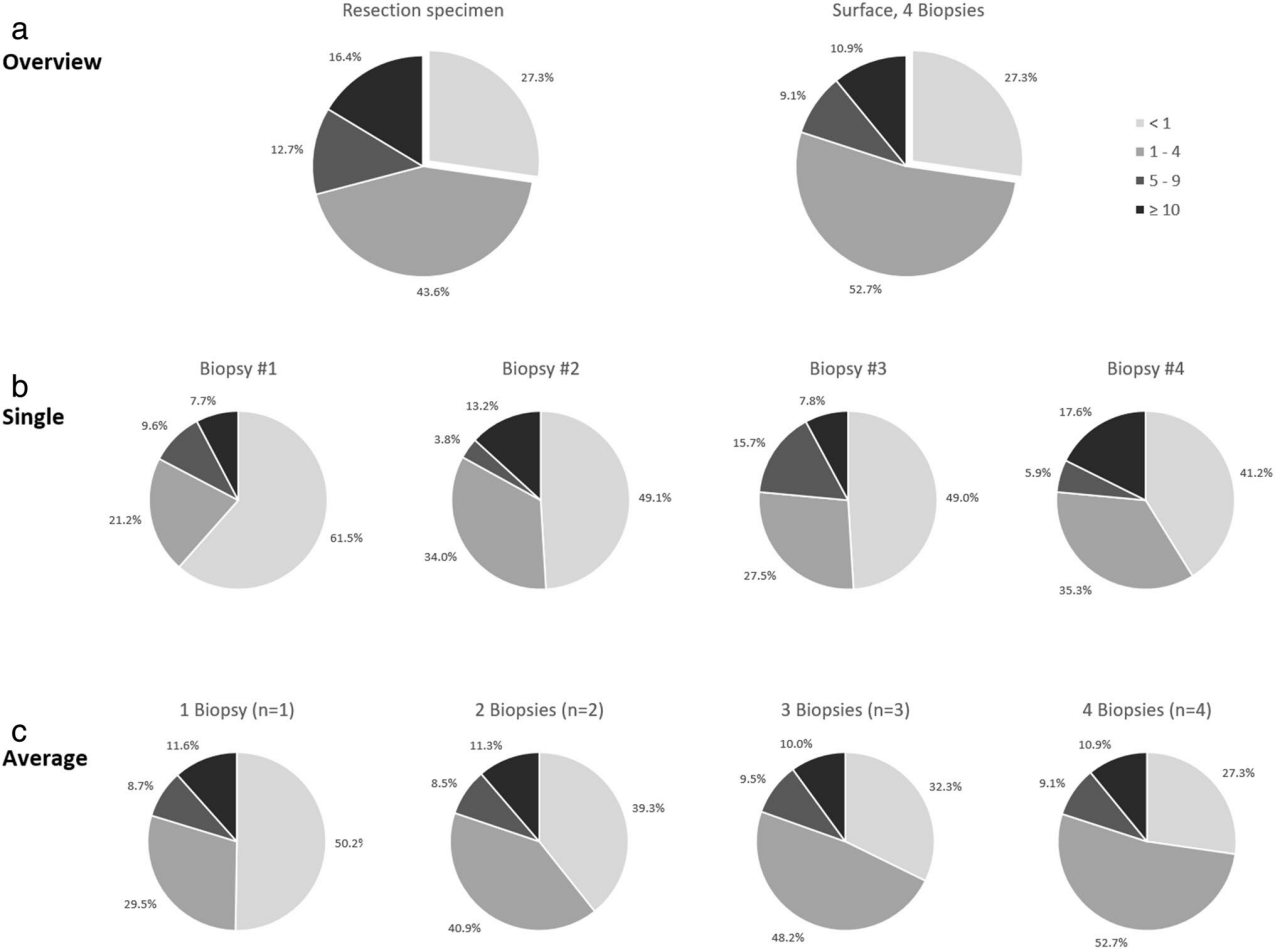
Distribution of PD-L1 expression in resection specimens and surface biopsies. **a**: Resection specimens vs. four biopsies (integrated by average score). **b**: Surface biopsies, single (one plot per biopsy). **c**: Surface biopsies, average of 1, 2, 3 and 4 biopsies

The PD-L1 scores of the resection specimens were regarded as true PD-L1 status of the cases and used as reference scores in the subsequent analyses. The resection specimens showed a roughly quartered PD-L1 distribution: About one quarter of cases was negative (CPS < 1; 27.3%), half of the cases showed low PD-L1 expression (CPS ≥ 1,  < 5; 43.6%) and one quarter showed moderate to strong expression (CPS ≥ 5, < 10; 12.7% and CPS ≥ 10; 16.4%) (Fig. 2a, SI –Fig. 2).

### Molecular subtypes and PD-L1 expression

The distribution of PD-L1 expression differed among the molecular subtypes. As expected, all MSI type tumors were PD-L1 positive (3/3, 100%). One out of two EBV-associated tumors were PD-L1 positive (CPS 5), one negative (CPS < 1).

### Preoperative diagnostic biopsies

In *n* = 22 cases, preoperative diagnostic biopsies were available. The biopsies contained 1–8 tissue particles (mean 3.5, standard deviation 1.9). Twelve cases contained less < 4 tissue particles. On average, 70% of the particles contained carcinoma cells (SI Fig. 4). More than half of the cases was PD-L1 negative (CPS < 1; 59.1%), 22.7% showed low PD-L1 expression (CPS ≥ 1, < 5) and about 9% showed moderate to strong expression (SI Fig. 5). Compared to the matched resection specimens, sensitivity was reduced to 0,412. Ten out of 22 preoperative biopsies are false negative.

## Results of TMAs/biopsies

Analyses of the TMAs/substitute-biopsies showed PD-L1 expression mostly in tumor-associated immune cells and marked heterogeneity in the majority of cases (Figs. 1, 2; SI Figs. 2, 3). About two-thirds of positive cases showed low expression levels (CPS ≥ 1, < 5), which was unevenly distributed among the four biopsies. More widespread expression was detected in the surface biopsies (Fig. 2) compared to the deep biopsies (SI Fig. 3).

If only one surface biopsy is analyzed per case, the prevalence of PD-L1 expression is reduced compared to the resection specimen: About one third of cases is PD-L1 positive (CPS ≥ 1) compared to three quarters (Fig. 2b). Sensitivity (0.57) is low while the specificity is high (0.9).

If several biopsies are analyzed, different approaches to interpretation are possible. Here, two ways were investigated: maximum CPS value and average CPS value.

- In the maximum value analysis, the highest CPS value of any of the four samples is used to classify the respective case. The resulting prevalence data strongly deviated from the reference scores and indicated frequent overestimation (data not shown). Thus, this approach was rejected.
- In the average CPS analysis, the arithmetic mean of the four samples is used to classify the sample. The four biopsies are thus evaluated combined. This approach showed good agreement with the scores of the resection specimens (Fig. 2).

Analysis of multiple surface biopsies increases the prevalence of PD-L1 positive cases as well as sensitivity and specificity (Fig. 2c). One, two, three and four biopsies per case were compared. Using four biopsies yielded best results: The prevalence showed a distribution similar to the resection specimens (Fig. 2a). In detail, the number of cases with CPS ≥ 1 was virtually similar; moderately and strongly positive cases were slightly reduced. Accordingly, sensitivity was 0.73 and specificity 1.0. If only two or three biopsies were used, the sensitivity dropped to 0.7 while the number of false-negative cases increased, including cases that were now classified as CPS < 1.

Deep biopsies of the invasion zone were less representative of the true PD-L1 status. Even four deep biopsies per case combined yielded only 46.5% positive cases (CPS ≥ 1) compared about three quarters if surface biopsies are used. Sensitivity was 0.5 and specificity 0.92 (SI Fig. 3).

## Discussion

PD-L1 immunohistochemistry is currently the only clinically approved predictive biomarker for immunotherapy. PD-L1 testing is performed by pathology laboratories using IHC assays and interpretation criteria defined in the approval documents of the respective therapeutic agents. In gastric cancer, most current studies assess PD-L1 with the 22C3 IHC-assay and combined positive score (CPS) interpretation. The PD-L1 CP-score correlates with an increased probability of a clinical benefit from PD-1 inhibition. In gastric cancer, CP-scores of ≥ 1 and ≥ 5 are associated with an increased probability of response to the PD-1 inhibitor Nivolumab (CheckMate 649 study, ESMO 2020).

The PD-L1 prevalence of the cases in this study are consistent with observations made in CheckMate 649 study concerning CPS ≥ 1: (72.7% our finding, vs. 82% CheckMate), although significantly fewer tumors with moderate or high PD-L1 positivity were found (CPS ≥ 5: 29.1% our finding vs. 60% CheckMate).Patient characteristics and molecular subtypes according to TCGA largely correspond to the expected distribution of a Western European patient population (Table 1). The proportion of microsatellite-unstable and EBV-positive tumors is slightly below the expected value (4.2% EBV and 10.5% MSI versus 9% and 22% in TCGA-collective) [19,20] Molecular subtype and PD-L1 prevalence were related and all MSI tumor were PD-L1 positive. One out of two EBV-associated carcinoma was also PD-L1 positive (CPS 5).

In this study, a PD-L1 LDT using antibody clone E1L3N was used. The LDT was calibrated to match the staining patterns of the 22C3 pharmDx assay. Several PD-L1 harmonization studies have demonstrated that E1L3N can be used to set-up 22C3 pharmDx-equivalent LDTs [21–23]. All PD-L1 assessors in this study were specifically trained for PD-L1 CP-scoring.

The modalities of PD-L1 assessment were not uniform among the different clinical trials (e.g., CheckMate 649 or Keynote 61 study). The PD-L1 status was determined either on therapy-naïve biopsies or on surgical specimens after neoadjuvant treatment. In some cases, biopsies of hematogenous metastases in different organs were tested. The proportion of available vital tumor tissue also varies from case to case. Thus, it is unclear how many tumor-bearing biopsies are necessary for reliable PD-L1 determination.

This is the first study systematically addressing the clinically relevant question of whether PD-L1 status assessed on endoscopically obtained biopsy material is indicative of the actual PD-L1 status of the entire tumor. The following question needed to be addressed:

- How is the spatial distribution of PD-L1 within a given tumor? Are PD-L1 positive cells possibly so heterogeneously distributed that biopsy material may not be able to represent the actual PD-L1 expression?
- If several biopsies are available, how should the interpretation be performed?

A homogenous patient cohort was tested that included only non-pretreated and primarily non-metastatic adenocarcinoma of the stomach.

The available preoperative diagnostic biopsies showed a reduced PD-L1 prevalence compared to the resection specimens and a reduced sensitivity.

TMAs of the resection specimen were constructed. Four TMA cores/biopsies were obtained from superficial, endoscopically accessible, non-necrotic tumor areas. TMA cores with a diameter of 1.2 mm correspond to the typical tumor cell content of an endoscopically obtained tumor biopsy. We assume that the chosen procedure reproduces a realistic clinical setting. To investigate spatial heterogeneity of PD-L1 expression, four additional biopsies were taken from the tumor depth, i.e., the level of deepest tumor infiltration. This area would not be reachable by endoscopic biopsies.

Comparisons of the CP-scores of the resection specimens and the TMA cores clearly demonstrate that surface biopsies are suitable to determine the true PD-L1 status. Surprisingly, TMA cores of the invasive front of the carcinoma were not representative and showed reduced sensitivity. However, an adequate number of surface biopsies is required to achieve valid results. Here, the combined analysis of four surface biopsies yielded a PD-L1 distribution similar to the resection specimen and best test characteristics with a sensitivity of 0.733. Using fewer biopsies reduced sensitivity and would result in false-negative results in a clinical setting. If just one tumor-bearing biopsy was analyzed, the proportion of positive cases (CPS ≥ 1) would drop to 49.8% while the sensitivity would be 0.566. The proportion of false-negative samples would increase to 30.8% compared to 18.1% for four biopsies. The preoperative biopsies also showed reduced PD-L1 prevalence and sensitivity, which is likely related to the limited number of tissue particles per case.

Biopsies from endoscopically accessible tumor areas are thus able to provide a largely realistic picture of the overall PD-L1 status if at least four tumor-bearing biopsies are available for analysis. Presumably, however, a higher tumor biopsy number is reasonable. The results are a clear plea for the requirement of a minimum number of four tumor-bearing, endoscopically obtained biopsies for the determination of therapy-relevant biomarkers. For the second therapy-relevant biomarker of gastric carcinoma, Her2/neu, a minimum of five tumor-bearing biopsies is also required for reasons of heterogeneous distribution of Her2/neu within the tumor. It is also stated that the risk of false-negative tumors increases if the number of tumor-bearing biopsies falls below five [24–26].

The results of this study should be verified in studies using larger patient’s cohorts, more than four biopsies and clinical response data. Here we used the PD-L1 scores of the resection specimens as true PD-L1 scores. However, the goal of PD-L1 IHC is prediction of response. Future studies should test the PD-L1 status of surface biopsies against the clinical benefit from PD-1 inhibition. Given the results of the present study, we would assume that more biopsies will improve the predictive value of PD-L1 testing in gastric cancer.

## Conclusion

Endoscopically obtained biopsies of gastric cancer can be indicative of the true PD-L1 status if sufficient material is sampled. In this first comprehensive study on PD-L1 assessment in gastric biopsies, an optimized procedure could be determined: If at least four biopsies with a total area of about 4.5 mm^2^ are sampled and analyzed combined, results similar to resection specimens may be obtained. The positivity rate is virtually similar with a specificity of 1.0; the number of highly positive cases (CPS ≥ 10) is slightly reduced, 10.9% vs. 16.4%; the overall sensitivity is 0.73.

## Supplementary Information

Below is the link to the electronic supplementary material.

Supplementary file1 (TIF 91 KB)

Supplementary file2 (TIF 17657 KB)

Supplementary file3 (TIF 826 KB)

Supplementary file4 (TIF 145 KB)

Supplementary file5 (TIF 31 KB)

Supplementary file6 (TIF 38 KB)
