# Supplementary figures and images for: Optimized PD-L1 scoring of gastric cancer
Source: Gastric Cancer. 2021 May 5;24(5):1115–22. doi: 10.1007/s10120-021-01195-4 (PMC8338825; doi:10.1007/s10120-021-01195-4)

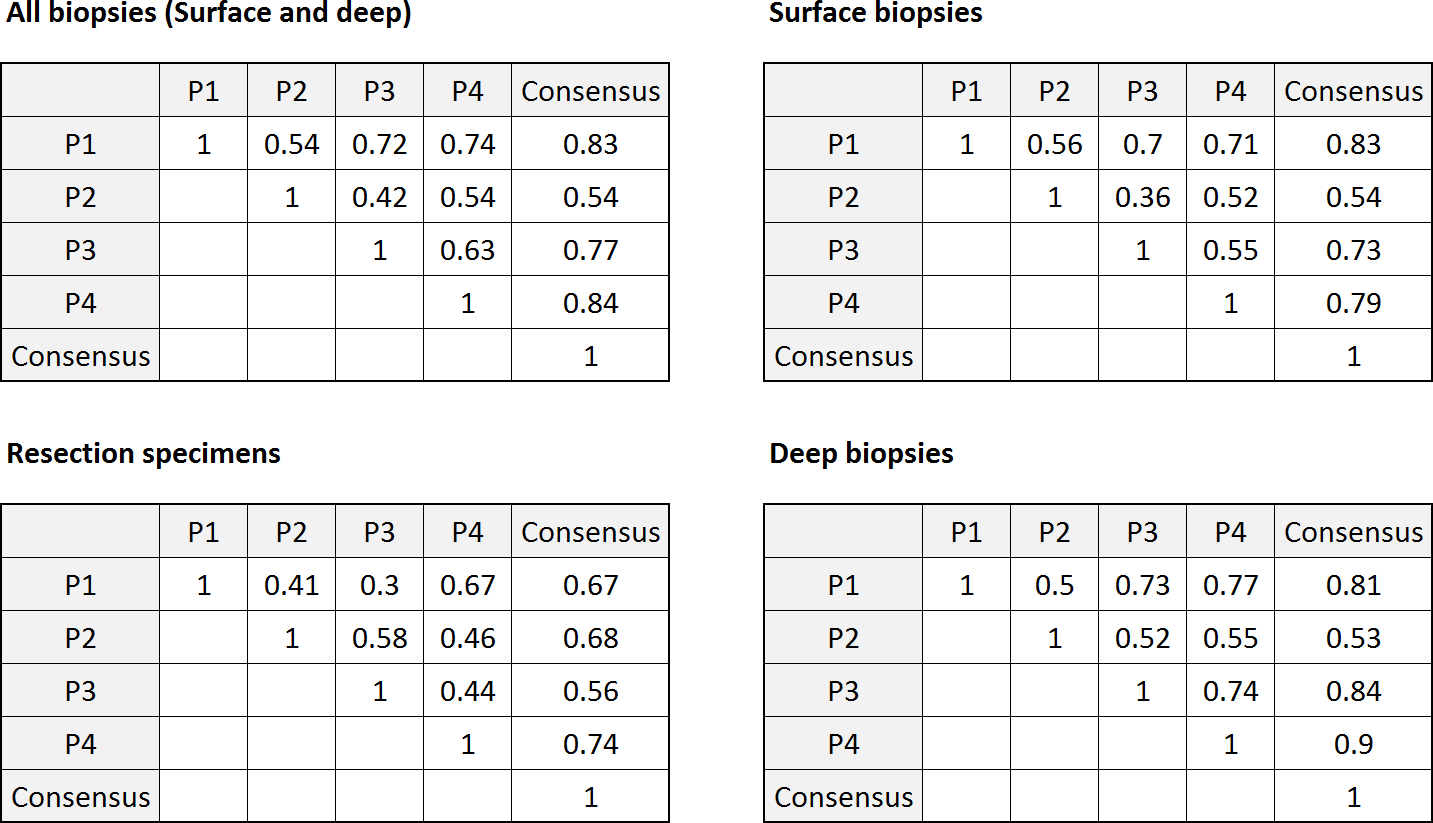

Supplement: Supplementary file 1 — Supplementary file1 (TIF 91 KB) [file 10120_2021_1195_MOESM1_ESM.tif]

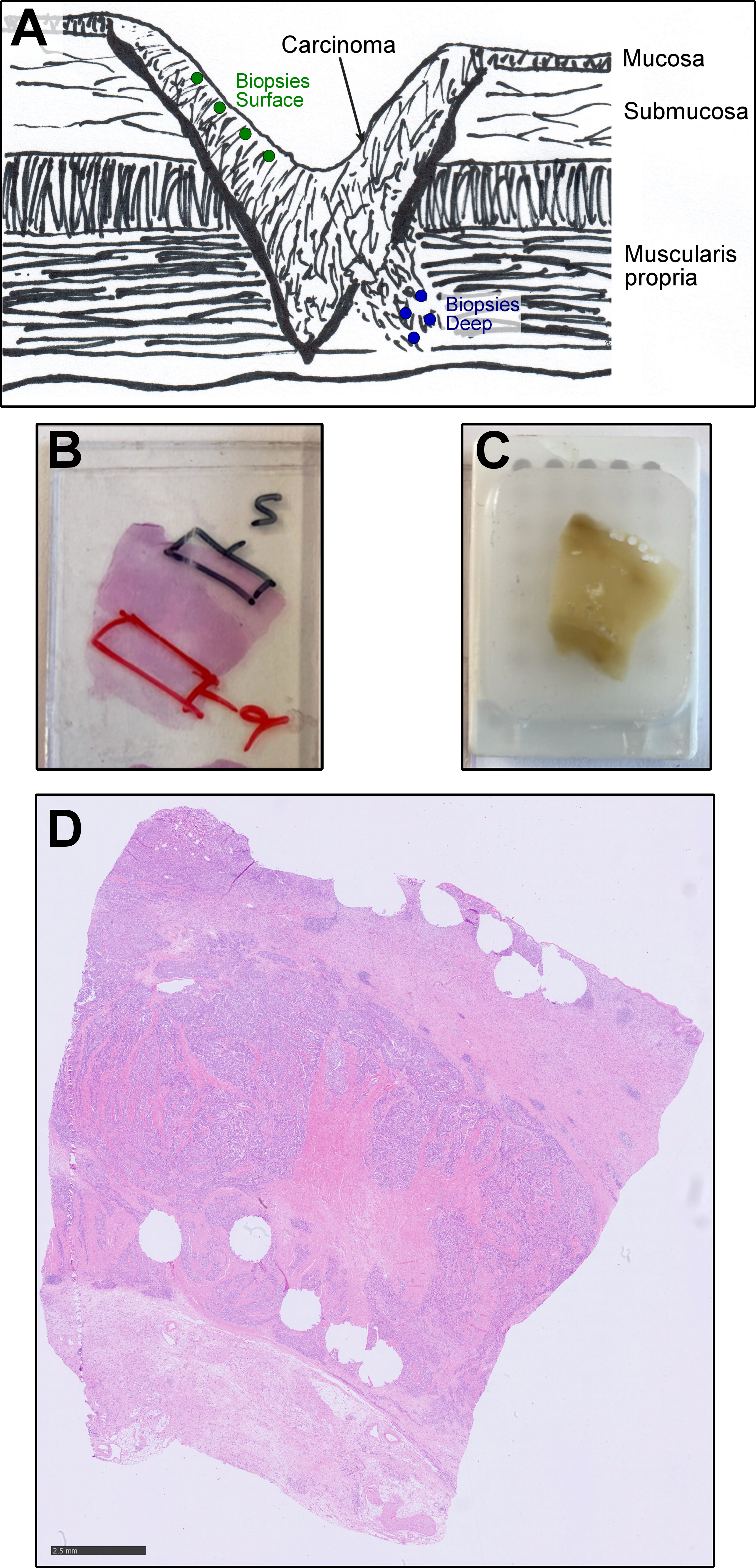

Supplement: Supplementary file 2 — Supplementary file2 (TIF 17657 KB) [file 10120_2021_1195_MOESM2_ESM.tif]

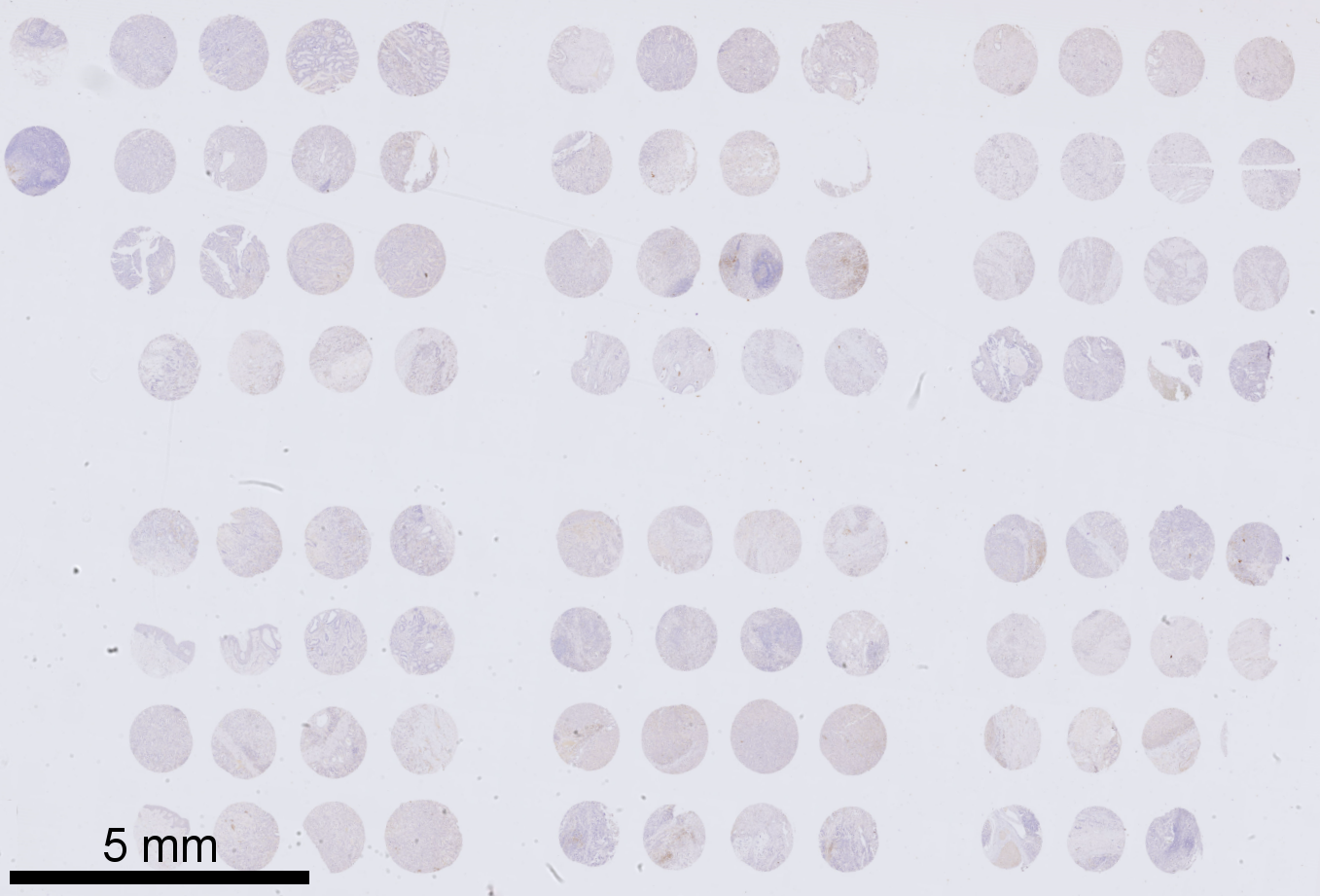

Supplement: Supplementary file 3 — Supplementary file3 (TIF 826 KB) [file 10120_2021_1195_MOESM3_ESM.tif]

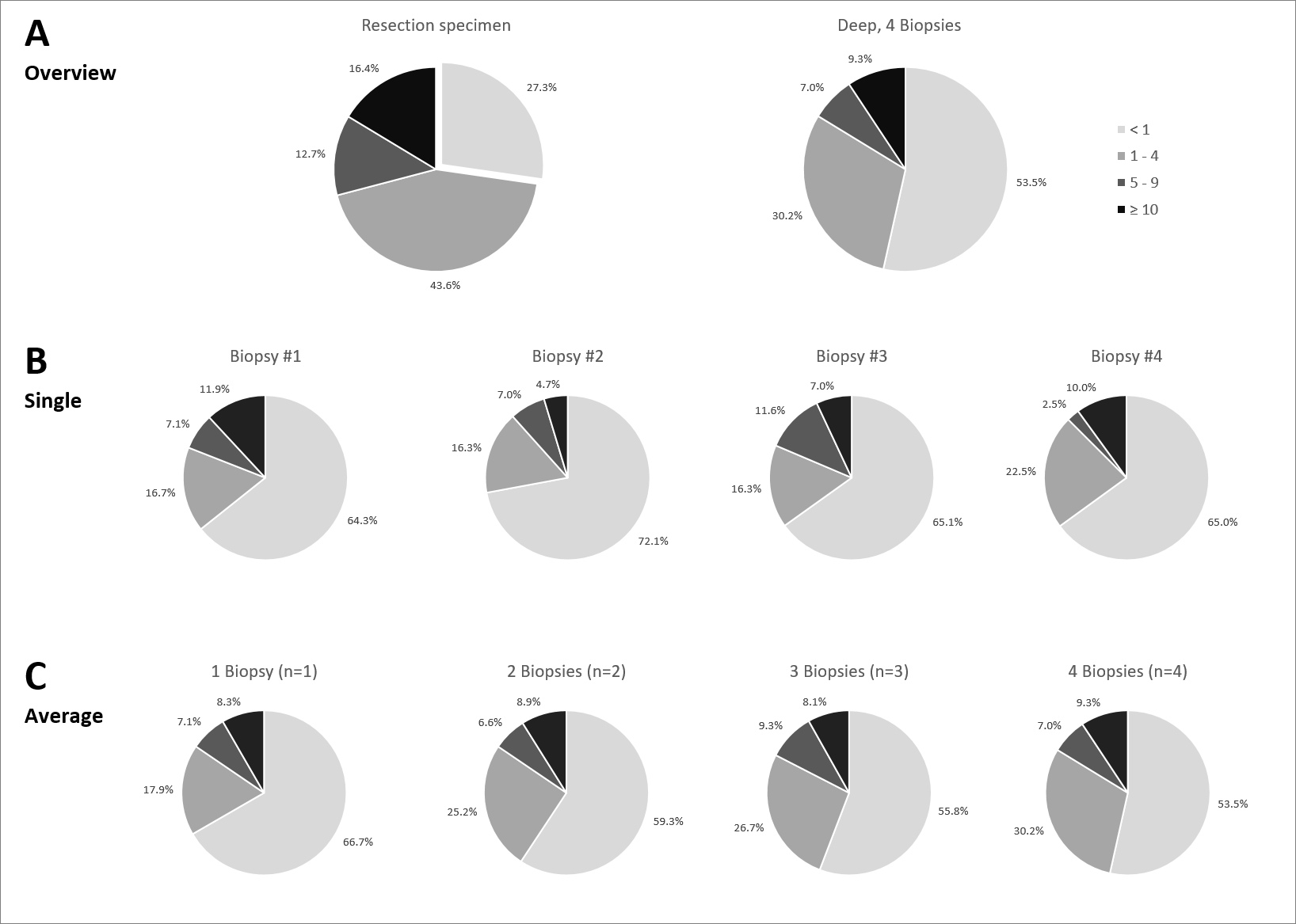

Supplement: Supplementary file 4 — Supplementary file4 (TIF 145 KB) [file 10120_2021_1195_MOESM4_ESM.tif]

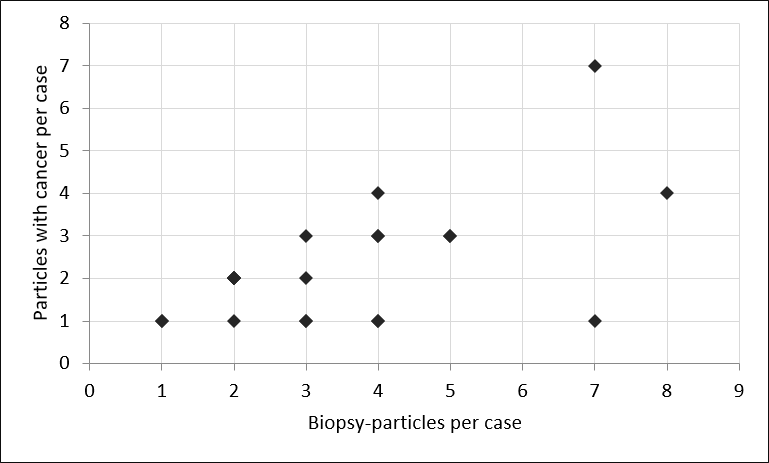

Supplement: Supplementary file 5 — Supplementary file5 (TIF 31 KB) [file 10120_2021_1195_MOESM5_ESM.tif]

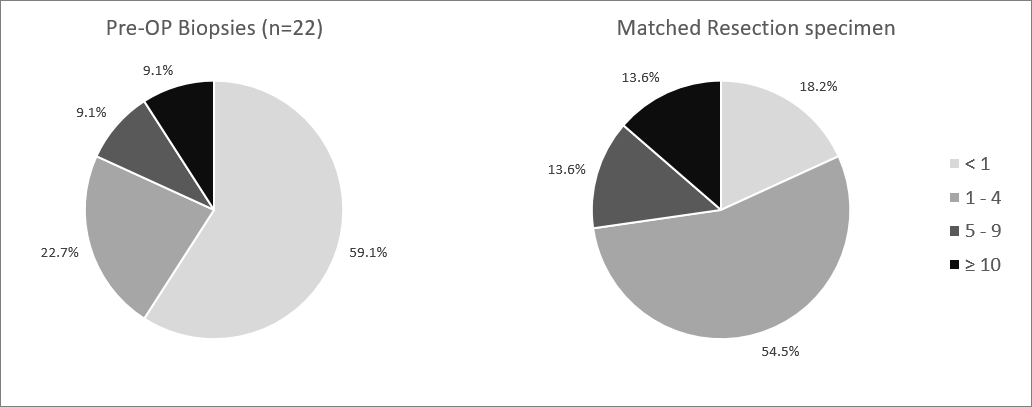

Supplement: Supplementary file 6 — Supplementary file6 (TIF 38 KB) [file 10120_2021_1195_MOESM6_ESM.tif]
